# Participants’ perspectives of weekly telephonic mood monitoring in South Africa: a feasibility study

**DOI:** 10.1186/s40814-018-0245-0

**Published:** 2018-02-22

**Authors:** A. S. J. Van der Watt, T. Roos, C. Beyer, S. Seedat

**Affiliations:** 1 Department of Psychiatry, Stellenbosch University, PO Box 19063, Francie van Zijl Drive, Cape Town, 7505 South Africa; 2 Department of Psychiatry, University of Cape Town, Anzio Rd, Cape Town, 7925 South Africa; 3 Department of Medicine and Allied Health Sciences, Stellenbosch University, Francie van Zijl Drive, Tygerberg, 7505 South Africa

**Keywords:** Anxiety disorder, Depression, Bipolar disorder, Experiences, Mood disorders

## Abstract

**Background:**

Mood and anxiety disorders have a high lifetime prevalence, and their chronicity adds to the management burden of already scarce and strained mental health care resources, particularly in developing countries. Non-professional-assisted interventions and technology (such as weekly telephonic mood monitoring) could assist in the early identification of symptoms of relapse and hospitalization prevention. The present study aimed to determine participants’ perspectives and the feasibility of weekly telephonic mood monitoring in order to inform the development of the full study.

**Method:**

Semi-structured telephonic interviews (*n* = 37; 89.2% female; mean age = 33.1 years) were conducted as part of the full-scale feasibility study (*N* = 61; named the Bipolar Disorder Mood Monitoring (BDMM) Study). The BDMM Study was conducted to determine the viability of weekly telephonic mood monitoring, spanning 26 weeks and starting 1 week post-discharge. Frequency and descriptive statistical analyses (using SPSS version 24) were undertaken, and qualitative data were analyzed using thematic content analysis.

**Results:**

This article presents the findings from the semi-structured interview section of the BDMM Study. Participants generally expressed positive experiences and perceptions of weekly telephonic mood monitoring, stating that they would advise others to also take part in weekly telephonic mood monitoring. Nonetheless, some participants did make suggestions for improvement of mood monitoring while others expressed negative experiences of weekly telephonic mood monitoring.

**Conclusion:**

The results of the semi-structured interviews of the BDMM Study indicated that participants perceived weekly telephonic mood monitoring to be helpful in lightening the burden of mood and anxiety disorders (e.g., having someone to talk to, providing insight into their disorders). Not only did it help them, but they also perceived mood monitoring to be potentially helpful to future participants. However, weekly mood monitoring was also burdensome in itself (including being too time consuming and having to answer questions when feeling down). Importantly, the findings highlighted that participants’ and researchers’ perceptions and experiences may not be congruent (especially in terms of therapeutic misconception). The current findings may inform researchers’ future approach to study design and participant relationships.

**Electronic supplementary material:**

The online version of this article (10.1186/s40814-018-0245-0) contains supplementary material, which is available to authorized users.

## Background

Mood (9.6%) and anxiety (12.9%) disorders are globally prevalent as shown in pooled data from 39 countries [1]. Additionally, the Global Burden of Disease Study highlighted the increasing challenge that these disorders pose for health systems, regardless of economic standing; with depressive disorders accounting for 40.5% and anxiety disorders for 14.6% of disability-adjusted life years (DALY) worldwide [2]. Adding to this burden is the increased frequency of relapses and subsequent hospitalizations, which are disruptive for individuals and their families and can lead to difficulties with work, housing, and the pursuit of meaningful life goals [3]. Conventional treatment methods, such as psychotherapy, are both expensive and time consuming [4].

Mental health care resources are scarce, especially in lower and middle-income countries (LMIC) [5], with up to 83% of people living in LMIC not receiving the mental health treatment they require [6]. Specifically, in South Africa, there are only 0.32 psychologists and 0.28 psychiatrists for every 100,000 people [7], in comparison to first world countries such as the USA with 29.03 psychologists and 7.79 psychiatrists per 100,000 [8]. Estimates indicate that the burden of mental and substance use disorders, in sub Saharan Africa, will increase by 130% by 2050 [9]. It is clear that without a different approach, this presents an overwhelming burden in already human-resource-starved contexts.

Non-professional-assisted interventions [10] and technology could help offset this burden by addressing the human resource deficit, while providing support for people suffering from mental illness [11]. Positive outcomes in LMICs have been demonstrated by studies that have utilized non-professionals to deliver mental health interventions [12]. One such intervention is distant inter-episodal mood monitoring of people suffering from mood and/or anxiety disorders for early identification of relapse, through detection of residual depressive symptoms [13] and providing support.

Studies using different methods of distant inter-episodal mood monitoring (including online web-based, text messaging, smart phone applications, and paper-and-pencil) were found to be both feasible and successful in augmenting treatment [14–17]. Additionally, studies reported high compliance and acceptance of regular mood monitoring [14, 15, 18]. However, the feasibility and effectiveness of routine mood monitoring in clinical care settings, through the use of brief rating scales administered by doctors or nurses, has not been examined in resource-limited contexts.

A recent South African study undertaken by the authors to determine the feasibility of inter-episodal mood monitoring found a high attrition rate (52.5% of 61 participants) despite the general acceptance of the study [19]. Little empirical mixed-method research has been conducted to determine participants’ perspectives of weekly telephonic mood monitoring. From a utilitarian perspective, an understanding of participants’ expectations and motivation can arguably inform researchers on how best to implement telephonic mood monitoring [20].

### Study aim and objectives

We aimed to determine participants’ perspectives and the feasibility of weekly telephonic mood monitoring to adapt the study design in order to improve retention for the full Mood Monitoring Study. The present article reports the findings of a specific part (i.e., the semi-structured interviews) of the Bipolar Disorder Mood Monitoring (BDMM) Study on the acceptability and perceived effectiveness of mood monitoring and the reasons provided by participants for dropping out.

## Methods

The semi-structured interviews were part of the BDMM Study of telephonic inter-episodal mood monitoring in participants with mood and/or anxiety disorders. The aim of the BDMM Study was to longitudinally track mood fluctuations in psychiatric hospital participants post-discharge. A detailed description of the BDMM Study is published elsewhere [19].

The semi-structured interviews were approached using the descriptive phenomenological framework [21] as influenced by the Husserlian philosophical phenomenological theory [22]. According to this theory, experiences that are perceived by human consciousness should be scientifically studied in order to understand human motivation, including the lived experience of phenomena and what a specific group of people perceive to be real (such as the lived experience of weekly telephonic mood monitoring by patients diagnosed with mood and/or anxiety disorders). Phenomenological research aims to describe a phenomenon without any pre-conceived ideas or prejudice while remaining true to the described experience [22]. Accordingly, the interviewers had little to no knowledge of the participants or of the existing literature on the experience of telephonic mood monitoring. They were thus able to approach the interview process without any preconceived ideas and/or prejudice. While phenomenological research holds that lived experiences are experienced uniquely by each individual [23], certain features of a phenomenon are shared by all people who experience it [21]. It is this shared experience of telephonic mood monitoring, as described within semi-structured interviews, that is reported in this article.

### Sampling and procedures

Registrars and psychiatric consultants at a governmental psychiatric hospital (in the greater Cape Town area) referred potential participants (patients diagnosed with mood and/or anxiety disorders) to the first author for recruitment. Thus, convenience sampling was used. The baseline assessment included a Demographic Questionnaire, the Childhood Trauma Questionnaire (CTQ), and the Life Events Checklist (LEC). After discharge, weekly mood fluctuations were telephonically assessed using the Quick Inventory of Depressive Symptomatology (QIDS-SR 6) and the Altman Self-Rating Mania Scale (ASRM), for 26 weeks.

A total of 61 participants were recruited into the BDMM Study to participate in a weekly telephonic mood monitoring feasibility study that spanned 26 weeks (starting 1 week post-discharge). The BDMM Study had a high dropout rate with 33 (54.1%) participants dropping out of the study. Of the 33 participants, 25 (75.6%) were identified as lost-to-follow-up after 3 consecutive weeks of missing data. Only seven participants actively voiced their desire to withdraw from the study. The majority of participants (59.4%) dropped out by week 9, with most participants (*n* = 7) dropping out at week 3. Fifteen cases of known re-hospitalization and six known suicide attempts were recorded during the study and reported to the Health Research Ethics Committee of Stellenbosch University (as part of the annual report), and, where known, the treating psychiatrists were informed of the attempted suicide. Every reasonable attempt was made to continue monitoring the mood of participants who were re-hospitalized.

As part of the BDMM Study, all participants were phoned (either at week 13 or 1 week post-discontinuation of the study) by two independent researchers (one post-graduate psychology student; one post-graduate neuropsychology student) to complete a semi-structured interview regarding their experience and perception of the study. However, only 37 (89.2% female; mean age = 33.1 years) of the 61 participants completed the semi-structured interviews. Interviews were not audio recorded; yet, notes were taken during the interviews and typed out once completed (see Additional file 1).

Table 1 presents the basic demographic and psychiatric data of the participants who have completed the semi-structured interviews (*n* = 37) and the full BDMM Study sample (*N* = 61).

**Table 1:** Participant demographic data and psychiatric diagnoses

Descriptive statistic	Semi-structured interviews (*n* = 37)		BDMM Study (total sample; *N* = 61)	
Age	Mean (SD) = 35.76 (10.8)		Mean (SD) = 35.3 (10.2)	
	Range = 18–53 years old		Range = 18–53 years old	
Highest level of education	Mode = Grade 12		Mode = Grade 12	
	Range = Grade 6—Honors		Range = Grade 6—MA	
Length of current admission (days)	Mean(SD) = 33.1(13.6)		Mean(SD) = 35.4(14.2)	
	*N*	%	*n*	%
Gender				
Male	4	10.8	14	23.0
Female	33	89.2	47	77.0
Ethnicity				
African/Black	0	0	2	3.3
Caucasian/White	16	43.2	27	44.3
Colored	19	51.4	31	50.8
Other	0	0	1	1.6
Marital status				
Single/no relationship	15	40.5	26	42.6
Relationship	7	18.9	8	13.1
Married	11	29.7	21	34.4
Divorced	3	8.1	5	8.2
Widowed	1	2.7	1	1.6
Employment				
Employed	12	32.4	19	31.1
Unemployed	25	67.6	42	68.9
Religion				
Christian	29	78.4	48	81.4
Muslim	2	5.4	3	4.9
Agnostic	6	16.2	7	11.5
Atheist	0	0	1	1.6
Not disclosed	0	0	2	3.3
Known substance use				
Yes, one	7	18.9	13	21.3
Yes, multiple	11	29.7	18	29.5
No	19	51.4	30	49.2
Mood and/or anxiety disorder				
Bipolar disorder	13	35.1	23	37.7
Major depressive disorder	21	56.8	32	52.5
Anxiety disorder	0	0	1	1.6
Mood and anxiety disorder	1	2.7	2	3.3
Other affective disorder^a^	2	5.4	3	4.9
Multiple psychiatric diagnoses				
Yes	14	37.8	25	41.0
No	23	62.2	36	59.0

^a^Substance use disorder with major depressive disorder as differential diagnosis, conversion disorder with major depressive disorder as differential diagnosis, schizoaffective disorder

The study was approved by the Human Research Ethics Committee of Stellenbosch University (S15/03/048). The study method was assessed using the COREQ [24] (see Additional file 2).

### Data analysis

Typed answers for all participants were combined to provide a descriptive summary. The first author carefully read through the answers to each question and identified quantifiable responses to each question. These were organized and collapsed into three to seven codes per question. Depending on the question, the codes (see Tables 2, 3, and 4 for codes to each question) included items such as:

(i) Negative and/or no;
(ii) Neutral (including “yes, but not really” and “not really, but yes”);
(iii) Positive and/or yes;
(iv) To help myself and/or others and/or the study;
(v) Apprehensive but it turned out good;
(vi) Surprise; or
(vii) Compliments to researcher/study and/or suggest changes.

**Table 2:** Study Participation

Question	*n*	%
Why did you decide to take part in the study?		
To help other patients or to help the study	9	24.2
To help myself	14	37.8
Because they asked and I was curious	9	24.3
To help other patients/the study, and myself	4	10.8
Other	1	2.7
Were there times that you felt like you did not want to continue with the study?		
No	21	56.8
No, but dropped out	6	16.2
Yes	9	24.3
Other	1	2.7
Will you be willing to take part in a similar research study again?		
No	2	5.4
Not sure, it depends	4	10.8
Yes	31	83.8
Would you advise others to take part in the study?		
No, I don’t know suitable people.	2	5.4
Yes	34	91.9
No answer given	1	2.7

**Table 3:** Study Acceptability

Question	*n*	%
How do you feel about the study in general?		
Neutral (okay; fine; alright)	9	24.3
Good (good; nice; great; positive; satisfied; it helps; interesting)	26	70.6
Non applicable answer given	2	5.4
How did you experience the day that the researcher first spoke to you about the study?		
Negative (hectic; shocking; nervous; uneasy; overwhelming; anxious)	4	10.8
Neutral (okay; fine)	3	8.1
Good (nice; comfortable; excited; professional; good; positive; open)	13	35.1
Don’t remember the day	2	5.4
Apprehensive but it turned out good	6	16.2
Surprised / unexpected / curious	4	10.8
Other	5	13.5
Would you change anything about the study?		
No	29	78.4
Doesn’t want to interfere	1	2.7
Yes	7	18.9
Do we ask questions that you do not like?		
No	34	91.9
Neutral (yes, but not really; not really, but yes)	3	8.1
Yes	0	0
Do you have any other comments and/or feedback regarding the study?		
No	15	40.5
Compliments to researcher/study	18	48.6
Compliments to researcher/study and suggest changes	2	5.4
Other	2	5.4

**Table 4:** Effectiveness and impact

Question	*n*	%
Do you think the questions we ask you every week are relevant?		
No	0	0
Neutral (yes, but not really; not really, but yes)	5	13.5
Yes	31	83.8
Non applicable answer given	1	2.7
What is it like to reflect about your mood on a weekly basis?		
Negative (doesn’t help; intense; draining; annoys me; takes time)	4	10.8
Neutral (on and off; difficult but helps; fine; too much but helps)	11	29.7
Good (it helps)	19	51.4
Other	3	8.1
Do you think this study will help other people who suffer from Mood Disorders?		
No	1	2.7
Yes, depending	6	16.2
Yes	30	80.1
Do you think this study helped you in any way?		
No	2	5.4
Neutral (don’t know; yes, but not really)	3	8.1
Yes	32	86.5

Participants’ answers were coded and recorded in an SPSS24 file. Frequency statistics were conducted to provide a general understanding and description of participants’ perspectives of mood monitoring.

Thematic analysis [25] was used to analyze the qualitative data. The first author re-read each participant’s typed semi-structured interview, noting emerging themes and rich quotations. Relevant quotations for each identified emerging theme were extracted and grouped together in a separate document. After coding the emerging themes of each individual transcription, transcriptions were combined and read as a whole. The initial primary codes were re-evaluated and collapsed where necessary. Additionally, rich quotations in support of quantitative findings were highlighted. The first author remained cognizant of her own knowledge and perceptions, noting these in a diary, in order to discern between the participants’ experiences and her own in order to ensure trustworthiness of the data analysis.

## Results

The semi-structured interviews formed a part of the BDMM Study, and the majority of the participants completed these interviews (*n* = 37; 60.7%). Nineteen of the participants phoned for the semi-structured interview could not be reached (31.1%), while five (5%) stated that they were not willing to complete the semi-structured interview. In terms of demographic information and psychiatric diagnoses, those who completed the semi-structured interview (*n* = 37) were comparable to the full BDMM Study sample (*N* = 61), with the exception of gender. Statistically, significantly more women completed the semi-structured interviews than those who did not (Fisher’s exact two-tailed *p* = 0.01).

Throughout the results section, participant quotations are referenced according to gender (male = M; or female = F), participation status (completed = C; or dropout = D), and participant number (for example FC-01). Three main themes and eight sub-themes emerged from the semi-structured interview data:

(i) BDMM Study participation
  - Reasons for deciding to participate in the BDMM Study
  - Reasons for wanting to withdraw from the BDMM Study
  - Willingness to take part in a similar study and to refer others to the BDMM Study
(ii) Study acceptability
  - Unacceptable negative experience of the BDMM Study
  - Initial surprise, hesitation, and uncertainty, but a good experience
  - An acceptable study requiring changes
(iii) Effectiveness and impact
  - The study helps and is applicable
  - The study helps you help yourself

### BDMM Study participation

Even though the dropout rate of the BDMM Study was high (52.5% of 61 participants), feedback provided by the participants in the semi-structured interviews did not generally reflect a wish to withdraw prematurely from the study (see Table 2). The majority indicated that they had not considered discontinuing, although the combined percentage of those who answered no but did drop out (16.2%) and those who answered yes (24.3%) was 40.1%.

#### Reasons for deciding to participate in the BDMM Study

The majority of participants indicated that they decided to participate in the BDMM Study (when approached in hospital) in order to receive help. This is indicated by the following quotations:

By me helping them I help myself (FC-36).

I thought it would get my mood right (FD-35).

I want to be healthy. I want to be stable. And I want to treat my family well. Therefore, I decided to take part in the study (FC-11).

Additionally, participants did not only want to help themselves or their immediate family but their fellow participants as well. This is indicated by participant FC-26 who said:

I decided to take part in this study, because I am struggling my entire life with depression and anxiety. When [Researcher] approached me it was the perfect opportunity to give feedback of my point of view about it. I participate not only to help myself but to help other patients suffering from mood disorders, so that doctors can offer better care [or] better mood monitoring during the early stages of the disorder, when it starts to develop. So that hopefully in the future the development of the disorder can even be prevented.

#### Reasons for wanting to withdraw from the BDMM Study

Reasons for wanting to withdraw prematurely included practical concerns of the study taking too long, the monotony of the study, and difficulty with combining the weekly phone calls with work schedules. This is indicated in the following quotations:

The study takes too long (FC-03).

[I wanted to withdraw] due to the same questions being asked over and over (FC-70).

[I withdrew] because I found work and I could not combine this study with work (FD-38).

Participants’ mood state also played a role in their participation and their consideration to withdraw from the study, as is evidenced by the following quotations:

I stopped because I was not feeling *lekker [good]* at that time. I was in low. I cut everybody out of my life. Therefore I decided to stop the study. Around that time I did not want to go to hospital (FD-35).

Yes [I felt like withdrawing]! Especially on the days when I was in a really bad mood or not having a good day then I didn’t want to talk to anyone (FC-56).

Sometimes I just don’t feel like talking at that specific time (FC-60).

#### Willingness to take part in a similar study and to refer others to the BDMM Study

The majority of participants were willing to take part in a similar study again and also indicated that they would advise others to take part in the BDMM Study as it would “help.” For example, participant FC-46 said: “I would encourage anyone with a similar problem to take part [in the BDMM study]”. However, other reasons were also provided for why the participants would take part in a similar study or refer others to take part in the BDMM Study. These reasons are indicated by the following quotations:

… just to be able to talk to someone … the people involved in the study and asking questions were very nice and helpful and it was better to open up and talk to them, than to anyone that I know (FD-29).

… [to talk to someone] who cared (FC-61).

… [to talk to someone] who seems to understand, since family doesn’t always understand you and the disorder … [it is an] opportunity to change things (FC-31).

### Study acceptability

Generally, participants expressed positive feelings about the study, saying that it was “good,” “fine,” and “nice” (see Table 3). However, some participants were also critical of the study. Participant FC-01, for example, indicated in the semi-structured interview that the BDMM Study was “nothing special.”

#### Unacceptable negative experience of the BDMM Study

Some participants mentioned that they had negative experiences of the study. For example, participant FD-35 said: “The study was too much for me.” In particular, participants also mentioned less than positive experiences of the baseline assessment (which included the two trauma questionnaires):

It was freaky and I was nervous. I didn’t know the people. I felt like a rat in a trial (FC-45).

The first time I have spoken to [Researcher] my mood was very bad … I have experienced it as very hectic, severe, and intense (FC-01).

I experienced the reality again, all blocked memories came back. It was shocking (FC-09).

In contrast to participant FC-09, participant FC-16 did not experience the recall of blocked memories as negative: “The conversation made me recall memories from the past. It was a good conversation.”

#### Initial surprise, hesitation, and uncertainty, but a good experience

Certain participants expressed their surprise at learning about the study. For example, participant FC-13 said: “I was shocked, since I thought caring would stop after the hospital. However, the opposite seems to be true” (FC-13).

Other participants indicated that they were initially hesitant to take part in the BDMM Study but that they then experienced that study as good, especially due to the support they received. This is illustrated by the following quotations:

I was quite apprehensive (FC-12).

[I was] hesitant at first, but afterwards I was fine (MD-30).

At first I was overwhelmed, but the students are doing a great job trying to help us (FC-52).

[I] didn’t know what to expect, but it turned out good (FC-56).

#### An acceptable study requiring changes

While the majority of the participants answered “no” when asked if they would change anything about the BDMM Study, throughout the interviews mention was made of aspects they would *like more* of. Notably, participants requested more advice and help from the researcher:

I would appreciate it to receive more encouragement and feedback about the problems I have. For instance, when a participant has a problem it would help if the researcher contacts the psychiatrist. I have been having problems to contact the psychiatrist myself. It would help if the researcher could be the link between the psychiatrist and me ... this study is one sided; the other side [hospital, researchers] does not help me … However, it would help if the researcher gives me some advice. For instance, I have low energy levels on a certain day, in this case it would help if the researcher advices me to take vitamin D tablets or to describe medicine (FC-10).

I feel in particular. This is because I receive no feedback [or] advice in this study. However, [Researcher] is very kind and encouraging. She advices me to go to the hospital in case necessary, but I do not receive any advice what I should do when I have a bad day or so (FC-01).

Participants also suggested shortening the study, making it less monotonous (for example changing the questions or phoning on different days), and making it more person specific.

### Effectiveness and impact

The majority (86.5%) stated that the study had helped them and had a positive impact on their lives (see Table 4). Notably, there was a distinction between perceiving the study to be helpful for others (external) and perceiving it to be beneficial to participants themselves (internal).

#### The study helps and is applicable

The semi-structured interviews indicated that participants perceived the BDMM Study to be pertinent to the experience of all mood disorder patients, as stated by participant FC-12: “It is applicable to the experience of all mood disorder patients.” Furthermore, participants believed it to be beneficial in terms of providing the doctors with knowledge which may assist them now and with future mood disorder patients, as illustrated below:

[It helps the doctors to] know how I’m feeling and give advice and stuff (FC-37).

The more feedback this study collects, the better the spectrum of the disorder is covered; this benefits future help [or] care (FC-26).

The doctor cannot diagnose your mood in a single session. A better diagnosis can be made when mood is monitored on a weekly basis. … more information is collected due to this research. It helps you foresee problems. But it could also offer better individual therapy. It could be a future way of diagnosing [or] tracking bipolar patients, since the mood monitoring collects more information so that better individual [or] personal therapy can be offered to a specific patient (FC-01).

#### The study helps you help yourself

Many participants found weekly mood monitoring to be a “helping tool” through which they learned to help themselves. For example, participant FC-28 said: “This [referring to the BDMM Study] holds you accountable to yourself.” Other participants also explained how the study helped them to take control of their own well-being:

It gives me a chance to improve myself. When I experience a lot of anger, I can sit back and reflect. This gives me the opportunity to better look after myself (FC-31).

It is a reflection of knowing how I have been and to see how I control it. It makes me monitor myself … the study shows me how strong I am. For instance, when I have a bad week and I want to cut myself; I can see what I have overcome via mood monitoring myself (FC-45).

I started to watch myself, especially alcohol intake, resulting in the fact that my symptoms improved (FC-12).

During mood swings, I learned myself to focus on the positive instead of focusing on the negative. I also know that during these times I need to sleep more thus I watch my sleep pattern (MD-21).

## Discussion

Semi-structured interviews, conducted as part of the BDMM Study, informed the understanding of the acceptability and perceived effectiveness of weekly telephonic mood monitoring. Additionally, these data provide a more nuanced understanding of the high dropout rate which is an important consideration for implementation of studies in the field (for the impact of mood monitoring on participants’ mood states, please see Van der Watt and colleagues [19]).

### The burden

Participants appeared to be cognizant of the burden of mood and anxiety disorders, not only the burden experienced by themselves, but also the *shared* burden with other psychiatric participants. They perceived weekly mood monitoring to be a way to lighten the burden through having a caring person to talk to. Prior research has also indicated the effectiveness of being listened to [26] or simply talking to a researcher who is interested in what they have to say [27]. Participants often take part in studies in order to obtain such therapeutic benefits [28]. Participants also perceived taking part in weekly mood monitoring as an opportunity to share necessary information with doctors (through study results being shared with doctors) that will enable better care for future participants. Altruistic motivation such as this has previously been identified as a motivating factor for participation in research studies [27, 28]. Additionally, similar to findings by McCusker and colleagues (2012) and Miklowitz and colleagues (2012), weekly mood monitoring was seen as a “helping tool” allowing participants to be accountable for their own mental health [29, 30].

In accordance with previous research by McCusker and colleagues (2012), participating in weekly mood monitoring was sometimes experienced as burdensome [29]. Aside from practical concerns, including being repetitive and time consuming, participants seemed to experience mood monitoring as especially arduous when their mood was “low” (FD-35). Others also noted a dislike for the impersonal (non-contact) nature of the study. Indeed, research by Miklowitz et al. [30] has documented high acceptance and compliance (88%) of weekly mood monitoring and mood management when personalized (including psychoeducation sessions to identify person-specific risk factors). It follows then that a more personalized mood monitoring system may increase study adherence and provide better health outcomes in general.

### Participants and researchers

Although approaching hospitalized patients to participate was a practical strategy from the researchers’ perspective—a recruitment approach commonly used in psychiatric research—participants experienced this differently. Some participants were surprised that “caring” (FC-13) would be continued post-discharge, and others were more apprehensive or felt like “a rat in a trial” (FC-45). This accords with previous research indicating initial anxiety and apprehension when approached to participate in research concerning emotive topics [27]. Although assessing participants’ trauma exposure may be important from a clinical and research perspective, some participants experienced it as “hectic” to recall memories. Lowes and Paul (2006) have also noted the concern of researchers that current distress may be exacerbated when discussing emotive or traumatic events.

There was also slight ambiguity regarding the role of the researcher (i.e., therapeutic misconception), with participants not able to distinguish the role of the researcher from doctors or therapists involved in their care. Some participants shared thoughts and feelings about their life or the previous week in relative detail and described the researcher as a personal psychologist, who provided weekly sessions. It is possible that the inherent power differential usually present during standard paper-and-pencil or application-based weekly mood monitoring was reversed when the researcher asked questions verbally, allowing for digression and human interaction. Participants arguably became experts on the topic (their own weekly lives) which allowed them to talk more freely and openly about personal and emotional issues [27, 31]. Consequently, even though the weekly mood monitoring was not intended to be therapeutic, catharsis appeared to be unavoidable and the weekly phone calls became a quasi-therapeutic experience [32].

There was a tendency where participants seemingly did not understand the role differentiation and bemoaned the fact that the researcher did not give advice as doctors/therapists do. However, it is also probable that participants (rightfully) assumed the researcher should have clinical knowledge and understanding of their mental illness, raising their expectations of both the study and the researcher beyond what is ethically and practically permissible [27]. Colbourne and Sque (2005) suggest that this need not be problematic and could indeed be conducive to the positive outcome of the researcher-participant relationship as long as it is acknowledged and its impact carefully considered.

Even though the same participants who were apprehensive or described the recruitment process as negative later credited the researchers for being nice and helpful, it is important to be cognizant of the influence of different experiences and perspectives not only on outcomes but also on participants’ relationship with the researcher. It is important for the researcher to build strong relationships with participants [28].

## Conclusion

Weekly telephonic mood monitoring was experienced as effective in lightening the shared burden of living with mood and/or anxiety disorders—whether through an internal or external locus of control. However, weekly telephonic mood monitoring with rating scales may also be impractical, burdensome, and challenging to implement in routine care, particularly in low resource settings. Thus, an understanding of patient insight, motivation, and concerns can be directly linked to withdrawal or retention in a study of this nature. Participants did not necessarily share the same perspectives as the researcher with regards to the recruitment process, the line of questioning, and the researcher’s role.

Taking the above into account, we have adapted the method for the full-scale study (which commenced October 2016) as listed below:

(i) We shortened the duration of weekly mood monitoring to 16 weeks.
(ii) We are cognizant of the repetitive, impersonal, and often “boring” nature of the questions, and encouraged continued participation, especially as the study draws to a close, without jeopardizing the integrity of data collection (e.g., asking the questions in a different order, asking the questionnaires in an alternating order, discussing clinically non-relevant benign topics with the patient to break the monotony).
(iii) Potential participants are briefed more thoroughly in advance by hospital staff (or their treating doctors) prior to being approached by research staff (e.g., the nature of the process, what to expect, what to do if you have concerns).
(iv) As part of the informed consent process, the researcher emphasized that she is not a clinician, in order to avoid incorrect expectations and possible disappointments and to mitigate issues of therapeutic misconception.

### Limitations

The following limitations should be considered when interpreting these findings. Firstly, the semi-structured interviews were not audio- or video-recorded, limiting the qualitative robustness of the data. The absence of data on non-verbal cues, which may have been insightful, potentially limits the contextual richness of the data and the accuracy of quotations. Additionally, even though every attempt was made to take thorough notes during the interviews (which were typed out), the lack of recordings limits the accuracy of the data. It is recommended that future studies include audio recordings in order to enhance the accuracy of the findings and the richness of the data.

Secondly, even though participants suggested changes to the study, they also indicated that they did not want to interfere with how researchers executed their work suggesting that they may not have been comfortable providing critique.

Finally, it is notable that the majority of participants in the present study were women (a combination of relatively more men dropping out of the study and/or being unwilling to conduct the semi-structured interview). It is possible that men are less receptive to weekly telephonic mood monitoring. However, gender differences in expectations and response to mood monitoring interventions deserve careful scrutiny.

## Additional files

Additional file 1: Semi-Structured Interview Schedule. (DOCX 22 kb)

Additional file 2: COREQ guiding questions and scoring / interpretation continued. (DOCX 26 kb)

## Abbreviations

ASRM: Altman Self-Rating Mania Scale
BDMM: Bipolar Disorder Mood Monitoring
C: Participant completed the study
CTQ: Childhood Trauma Questionnaire
D: Participant dropped out of the study
DALY: Disability-adjusted life years
F: Female
LEC: Life Events Checklist
LMIC: Lower and middle-income countries
M: Male
QIDS: Quick Inventory of Depressive Symptomatology
