# Supplementary material for: Participants’ perspectives of weekly telephonic mood monitoring in South Africa: a feasibility study
Source: Pilot Feasibility Stud. 2018 Feb 22;4:56. doi: 10.1186/s40814-018-0245-0 (PMC5824463; doi:10.1186/s40814-018-0245-0)
Supplement: Supplementary file 1 — Semi-Structured Interview Schedule. (DOCX 22 kb) [file 40814_2018_245_MOESM1_ESM.docx]

# Additional file 1

## Semi-Structured Interview Schedule

Please answer the following questions as truthfully as possible. Please also elaborate on your answers wherever possible and as much as possible.

1. How do you feel about the study in general?
2. How did you experience the day that the researcher first spoke to you about the study?
3. Is what you expected the study to be like the same as you experience it now? If not, what is different?
4. If you answered “no” to the above question, is the difference a *good* or a *bad* difference and why?
5. Would you change anything about the study and if so, what and why?
6. Do you think the questions we ask you every week are relevant and why?
7. Do we ask question that you do not like and why?
8. If you answered yes to the above questions, please provide examples of the questions you do not like.
9. What is it like to reflect about your mood on a weekly basis?
10. Were there times that you felt like you didn’t want to continue with the study? If so, when and why?
11. Why did you decide to take part in the study?
12. Do you still have the same reason for taking part in the study? If no, what is your reason now?
13. Would you advise others to take part in the study and why?
14. Do you think this study will help other people who suffer from Mood Disorders and why?
15. Do you think this study helped you in any way and why?
16. Will you be willing to take part in a similar research study again?
17. Do you have any other comments and/or feedback regarding the study
