# Supplementary material for: Participants’ perspectives of weekly telephonic mood monitoring in South Africa: a feasibility study
Source: Pilot Feasibility Stud. 2018 Feb 22;4:56. doi: 10.1186/s40814-018-0245-0 (PMC5824463; doi:10.1186/s40814-018-0245-0)
Supplement: Supplementary file 2 — COREQ guiding questions and scoring / interpretation continued. (DOCX 26 kb) [file 40814_2018_245_MOESM2_ESM.docx]

# Additional file 2

## COREQ guiding questions and scoring / interpretation continued

|  | **Guiding Question** | | | | **Scoring / Interpretation** |
| --- | --- | --- | --- | --- | --- |
| **1** | Which author(s) conducted the interview or focus group? | | | | 0 = No disclosure  1 = Non-specific disclosure  **2 = Full disclosure - Specific name provided in Acknowledgements** |
| **2** | What were the researcher’s credentials? E.g. PhD, MD etc. | | | | 0 = No disclosure  1 = Non-specific disclosure  **2 = Full disclosure – Indicated in Data Collection section.** |
| **3** | What was their occupation at the time of the study? | | | | 0 = No disclosure  1 = Non-specific disclosure  **2 = Full disclosure – Indicated in Data Collection section.** |
| **4** | Was the researcher male or female? | | | | 0 = No disclosure  **1 = Disclosed – Evident in their names provided in Acknowledgements** |
| **5** | What experience or training did the researcher have? | | | | 0 = No disclosure  **1 = Non-specific disclosure**  2 = Full disclosure |
| **6** | Was a relationship established prior to study commencement? | | | | 0 = No disclosure  1 = Non-specific disclosure  **2 = Full disclosure – States as “independent” in Data Collection section** |
| **7** | What did the participants know about the researcher? E.g. personal goals, reasons for doing the research etc. | | | | 0 = No disclosure  **1 = Full disclosure – Reasons for conducting the research was explained during the informed consent process (as indicated in Declarations section as part of the Ethics Statement).** |
| **8** | | What characteristics were reported about the interviewer / facilitator? E.g. bias, assumptions, reasons and interests in the research topic. | | 0 = No disclosure  **1 = Non-specific disclosure – Theoretical framework was used**  2 = Full disclosure | |
| **9** | | What methodological orientation was stated to underpin the study? E.g. grounded theory, discourse analysis, ethnography, phenomenology, content analysis etc. | | 0 = No disclosure  1 = Non-specific disclosure  **2 = Full disclosure – Clearly indicated as using the descriptive phenomenological framework** | |
| **10** | | How were participants selected? E.g. purposive, convenience, consecutive, snowball | | 0 = No disclosure  **1 = Full disclosure - Convenience** | |
| **11** | | How were participant approached? E.g. face-to-face, telephone, mail, email. | | 0 = No disclosure  **1 = Full disclosure – At governmental psychiatric hospital, face-to-face; and telephonically.** | |
| **12** | | How many participants were in the study? | | 0 = No disclosure  **1 = Full disclosure** | |
| **13** | | How many people refused to participate or dropped out? Reasons? | | 0 = No disclosure  1 = Non-specific disclosure  **2 = Full disclosure – See “Feasibility Study Adherence”** | |
| **14** | | Where was the data collected? E.g. home, clinic, workplace etc. | | 0 = No disclosure  **1 = Full disclosure - Telephonically** | |
| **15** | | Was anyone else present besides the participants and researchers? | | **0 = No disclosure**  1 = Non-specific disclosure  2 = Full disclosure | |
| **16** | | What are the important characteristics of the sample? E.g. demographic data etc. | | 0 = No disclosure  1 = Non-specific disclosure  **2 = Full disclosure – See “Semi-Structured Interview Sample”** | |
| **17** | | Were questions, prompts, guides provided by the authors? Was it pilot tested? | 0 = No disclosure  1 = Non-specific disclosure  **2 = Full disclosure – See “Appendix 1”** | | |
| **18** | | Were repeat interviews carried out? If yes, how many? | **0 = No disclosure**  1 = Non-specific disclosure  2 = Full disclosure | | |
| **19** | | Did the research use audio or visual recording to collect the data? | 0 = No disclosure  **1 = Field notes / researcher notes only – See “Data Collection”**  2 = Recordings | | |
| **20** | | Were field notes made during and/or after the interview or focus group? | 0 = No disclosure  **1 = Yes – See “Data Collection”** | | |
| **21** | | What was the duration of the interviews or focus groups? | **0 = No disclosure**  1 = Full disclosure | | |
| **22** | | Was data saturation discussed? | **0 = No disclosure – NA as interview is to be conducted with every participant, regardless of saturation**  1 = Non-specific disclosure  2 = Full disclosure | | |
| **23** | | Were transcripts returned to participants for comment and/or correction? | **0 = No disclosure**  1 = Non-specific disclosure  2 = Full disclosure | | |
| **24** | | How many data coders coded the data? | | | 0 = No disclosure  **1 = Full disclosure – First author. See “Qualitative Analysis”** |
| **25** | | Did authors provide a description of the coding tree? | | | **0 = No disclosure**  1 = Full disclosure |
| **26** | | Were themes identified in advance or derived from the data? | | | 0 = No disclosure  **1 = Full disclosure – See “Qualitative Analysis”** |
| **27** | | What software, if applicable, was used to manage data? | | | 0 = No disclosure  **1 = Full disclosure – See “Quantitative Analysis”** |
| **28** | | Did participants provide feedback on the findings? | | | **0 = No disclosure**  1 = Non-specific disclosure  2 = Full disclosure |
| **29** | | Were participant quotations presented to illustrate the themes/findings? Was each quotation identified? E.g. participant number. | | | 0 = No quotations given  1 = Non-specific disclosure  **2 = Full disclosure – See “Results”** |
| **30** | | Was there consistency between the data presented and the findings? | | | 0 = No quotations given or no consistency of quotations given  1 = Non-supportive quotations  **2 = Supportive quotations – See “Results”** |
| **31** | | Were major themes clearly presented in the findings? | | | 0 = Not clearly presented  **1 = Clearly presented** |
| **32** | | Is there a description of diverse cases or discussion of minor themes? | | | 0 = Not clearly presented  **1 = Clearly presented** |
